# The *C. elegans* Homolog of RBBP6 (RBPL-1) Regulates Fertility through Controlling Cell Proliferation in the Germline and Nutrient Synthesis in the Intestine

**DOI:** 10.1371/journal.pone.0058736

**Published:** 2013-03-11

**Authors:** Ping Huang, Xuan Ma, Yanmei Zhao, Long Miao

**Affiliations:** 1 Laboratory of Noncoding RNA, Institute of Biophysics, Chinese Academy of Sciences, Beijing, China; 2 University of Chinese Academy of Sciences, Beijing, China; University of North Carolina at Chapel Hill, United States of America

## Abstract

RBBP6 (retinoblastoma binding protein 6, also known as PACT or P2P-R in humans) is a multi-domain protein that functions in multiple processes, such as mitosis, cell differentiation, and cell apoptosis. RBBP6 is evolutionarily conserved and is present in unicellular organisms to mammals. Studies of RBBP6 have mostly focused on its RB- and p53-binding domains, which are found exclusively in mammals. Here, we investigated the *C. elegans* homolog of RBBP6 to explore the functional roles of its other domains. We found that RBPL-1, the homolog of RBBP6 in *C. elegans*, is indispensable for worm development. RNAi silencing of *rbpl-1* led to embryonic lethality, as well as defects in oocyte production and intestine development. *rbpl-1* RNAi worms showed defects in germ cell proliferation, suggesting that RBPL-1 regulates mitosis. Moreover, RNAi silencing of *rbpl-1* inhibited nutrient synthesis in the worm intestine. RBPL-1, as a nucleolus protein, was found to be expressed in diverse tissues and necessary for both germline and soma development. Using microarray analysis, we identified ≈700 genes whose expression levels were changed at least 10-fold in *rbpl-1* worms. We propose that RBPL-1, like its yeast homolog, may regulate gene expression as an mRNA cleavage and polyadenylation factor. Taken together, the findings from this study reveal that RBPL-1 plays a pivotal role in *C. elegans* germline and soma development, suggesting that the functions of RBBP6 are conserved in diverse eukaryotic species.

## Introduction

RBBP6 was first identified in mouse testis and was implicated in controlling cell proliferation and differentiation [1]. It is an evolutionarily conserved 250-kDa multi-domain protein present in a wide variety of eukaryotic organisms ranging from microsporidia, to plants, to vertebrates. RBBP6 is composed of six domains–a DWNN, zinc knuckle, RING finger, SR, Rb, and a p53-binding domain–in vertebrates [2], while its homologs in all other species lack the Rb and p53-binding domains. RBBP6 controls a wide range of biological processes, such as mitosis, mRNA processing, ubiquitination, and translation [1], [2], [3], [4], [5]. It is also considered to be a putative E3 ligase owing to the presence of its RING finger domain [2]. In humans, the molecular mechanisms underlying its function depend on its ability to bind Rb and p53 to mediate their degradation [6], [7]. There are three splice variants of RBBP6 in mouse, called P2P-R, PACT, and RBQ-1 [5], [6], [8]. P2P-R was shown to localize to the nucleolus of interphase cells and the periphery of chromosomes in cells undergoing mitosis [9]. RBBP6 was also recently proposed to be a U-box protein with a role in controlling protein degradation [10].

Most studies of RBBP6 have been performed on human and mouse cells, because of its ability to regulate the p53 pathway and prevent tumorigenesis, and thus may be a potential target for cancer therapy [6], [7], [11]. However, compared with mammalian RBBP6, RBBP6s in other species that lack the Rb and p53-binding domains are not well studied [2]. In the present study, we identified the sole homolog of mammalian RBBP6 in *C. elegans*, RBPL-1, and characterized the roles it plays in *C. elegans* development. RNAi for *rbpl-1* resulted in defects in oocyte production and inhibition of intestine development. We show that RBPL-1 regulates nutrient synthesis in the intestine and germ-cell proliferation, which is reminiscent of its mammalian homolog P2P-R who is involved in mitosis. We also show that RBPL-1 is ubiquitously expressed in a variety of tissues and localizes to the nucleolus. Furthermore, using microarray analysis, we show that there are ≈700 genes whose expression levels are changed by at least 10-fold upon silencing of RBPL-1. Finally, we propose that RBPL-1, like its homolog in yeast [12], controls gene expression as an mRNA cleavage and polyadenylation factor.

## Materials and Methods

### *C. elegans* Strains and Growth Conditions

C. elegans were manipulated and cared for using standard techniques [13]. The worms were cultured at 20°C and derived from the N2 strain unless indicated. VC684 (*tag-214*), N2, DR466 (*him-5*), NL2098 (*rrf-1*), CB4856, NR222, AZ212, OD95 were obtained from the Caenorhabditis Genetics Center. VP303 was kindly provided by Kevin Strange (Mount Desert Island Biological Lab). *bIs1* (vit-2::GFP) was kindly provided by Xiaochen Wang (National Institute of Biological Sciences).

### RNAi Experiments

The RNAi assay was performed as described in [14]. RNAi bacteria were obtained from the Ahringer library [15]. Sequencing confirmed the *tag-214* RNAi clone contained the partial *tag-214* gene (start/end sequence: agtttcggaacgagggtttt….gtagatgatccggacgcaat). Bacteria containing the empty vector L4440 were used as a control. Eggs were placed directly onto RNAi plates containing 100 µg/L ampicillin and 1 mM IPTG. Reverse transcription polymerase chain reaction (RT-PCR) was used to verify the RNAi efficiency.

### Nile Red Lipid Staining and Fluorescence Imaging

A modified version of the staining performed in [16] was used in this experiment. About 100 adult nematodes were collected in 1 ml of water. 50 µl of freshly prepared 10% paraformaldehyde solution was added and mixed. Then the worms were frozen in liquid nitrogen, and thawed three times. The worms were allowed to settle, and the paraformaldehyde solution was removed. 1 ml of 1 µg/ml Nile Red in M9 was added to the worms and incubated for 30 minutes at room temperature with occasional, gentle, agitation. Worms were allowed to settle, washed, until most of the staining solution was removed. Finally, the worms were examined by epifluorescence microscopy (Axio Imager M2, Carl Zeiss), and the lipid content was quantified based on fluorescence intensity using Image J software (National Institutes of Health).

### Immunofluorescence

Adult hermaphrodite worms were anesthetized, and the gonads were dissected on coverslips. The cover slide and sample was immediately immersed into liquid nitrogen followed by the cold methanol treatment. Then, the coverslip was washed with PBST (PBS containing 0.1% Triton) and incubated with the anti-P-H3S10 antibody (1∶1,000 dilution with 5% BSA in PBST). The coverslip was washed with PBST and incubated with secondary antibody (1∶10,000 dilution with 5% BSA). The sealed coverslip (the seal solution contained DAPI) was observed under an epifluorescence microscope, and the mitosis ratio (the number of cells undergoing mitosis (marked by anti-P-H3S10) divided by the total number of cells (marked by DAPI) in the mitotic zone) was calculated.

### Life Span Assay

At least 50 eggs from each strain were placed onto RNAi plates. When the worms had grown to the young adult stage, they were transferred to new plates daily and the number of surviving worms was counted.

### Generation of the GFP-tagged RBPL-1 Transgenic Line

A strain carrying the transgenic arrays was constructed using standard methods [17]. The promoter and coding sequence of RBPL-1 were amplified from genomic DNA and cloned into the expression vector pPD95.77. Transgenic worms were generated by microinjection with 50 ng/µL gene vector mixed with 50 ng/µL marker vector pRF4. Transgenic worms were subjected to γ-ray radiation to obtain a stable line. Three stable lines were acquired, and all the three strains had the same fluorescence expression pattern. The worm line with the strongest fluorescence intensity was back-crossed to the N2 strain five times. Then, RBPL-1::GFP worms were crossed with OD95 worms to generate a strain with GFP labeled membranes and mCherry labeled histone H2B in addition to the RBPL-1 GFP expression.

### Microarray Assay

RNA was extracted from the eggs of RBPL-1 RNAi and control worms using Trizol reagent (Invitrogen, Carlsbad, CA, USA). RNA samples were sent to Shang Hai Biochip Company for further processing and microarray assays.

## Results

### RBPL-1, the *C. elegans* Homolog of Mammalian RBBP6, Regulates Fertility

**Figure 1:**
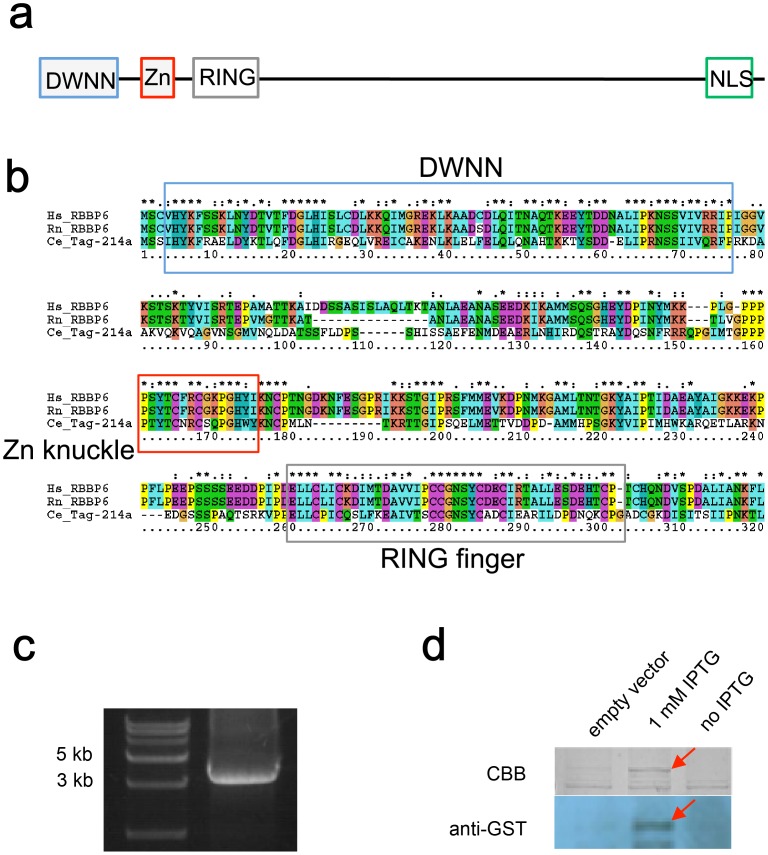
RBPL-1 is conserved in *C. elegans*. a. Predicted structure of RBPL-1 (TAG-214). The conserved domains DWNN, zinc knuckle, and RING finger are indicated by blue, red, and gray boxes, respectively. The nuclear localization signal (NLS) is indicated by a green box. **b.** Protein sequence alignment highlights the conservation of the DWNN, Zn knuckle, and RING finger domains among human, rat, and *C. elegans* RBBP6 proteins. **c.** Full-length *rbpl-1* mRNA cloned by RT-PCR. **d.** RBPL-1 expressed in *E. coli*. Upper: SDS-PAGE analysis of the bacterially expressed GST-RBPL-1 fusion protein, indicated by the red arrow. Lower: western blotting analysis of GST-RBPL-1 fusion protein (the red arrow) using an anti-GST antibody.

To identify the *C. elegans* homolog of mammalian RBBP6, we first performed a BLAST analysis using human and rat RBBP6s against the *C. elegans* genome. This analysis identified tag-214 (F36F2.3) as the only RBBP6 homolog in *C. elegans*. Tag-214 shares the highly conserved DWNN, zinc knuckle, and RING finger domains with mammalian RBBP6s (Figure 1A, 1B); therefore, we designated tag-214 as RBPL-1 (retinoblastoma-binding-protein-like-1). Like the *Drosophila* and *Arabidopsis* RBBP6 homologs, RBPL-1 lacks the SR, Rb and p53 binding domains that are exclusively found in mammals [2]. RBPL-1 has two splice variants (a and b). Variant a (3,367 bp) encompasses variant b (1,092 bp); therefore, our subsequent analyses focused on variant a. By RT-PCR, we confirmed the expression of the full-length *rbpl-1* transcript in N2 worms (Figure 1C). Expression of GST-tagged RBPL-1 in *E. coli* produces a 180-kDa band, which was verified to be RBPL-1 by western blotting using an anti-GST antibody (Figure 1D) and mass spec analysis (data not shown).

**Figure 2:**
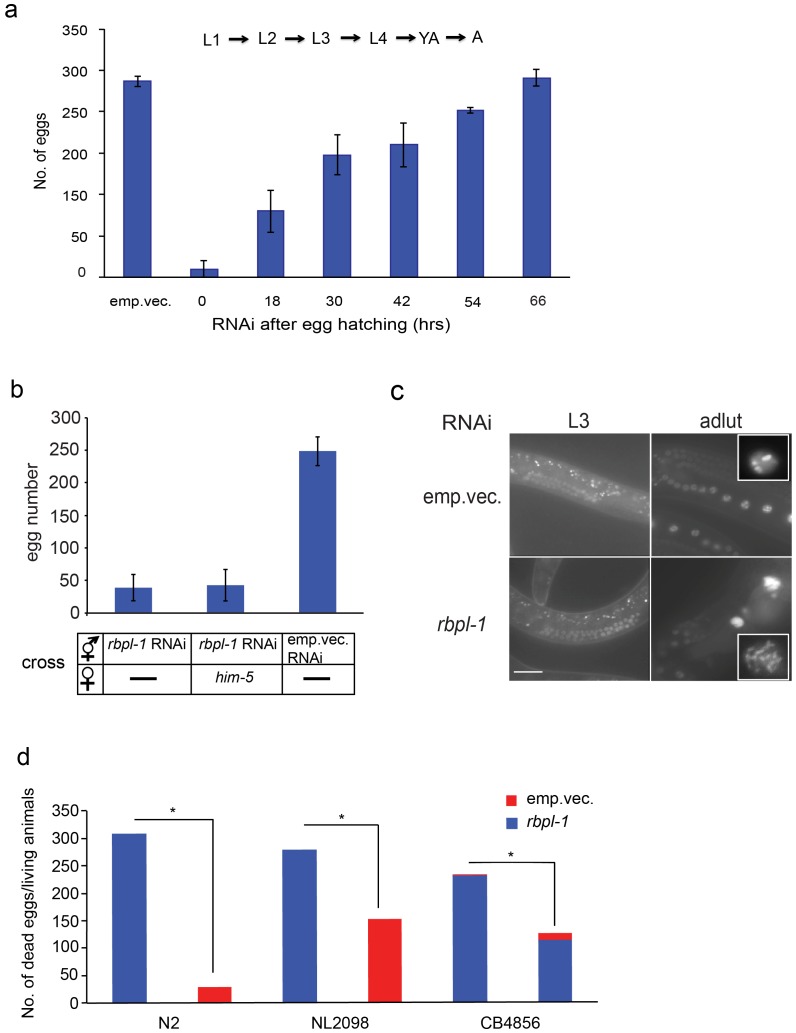
RNAi for *rbpl-1* reduces oocyte number. a. RNAi knockdown of *rbpl-1* results in reduced embryo number. The earlier RNAi is applied, the more severe the defects. The empty vector L4440 was used as an RNAi control. The grow temperature was 15°C. (YA = Young Adult, A = Adult). **b.** *rbpl-1* RNAi silenced hermaphrodites crossed with *him-5* males produce no more progeny than control RNAi hermaphrodites only. **c.** Adult *rbpl-1* RNAi worms show abnormal germline development. There is no difference between the *rbpl-1* RNAi and control RNAi in germline development at L3 stage. In adults, the oocytes are abnormal and reduced in *rbpl-1* worms. Some oocytes in *rbpl-1* RNAi adult possess multiple sets of chromosomes (shown in the inset) (H2B::GFP fluorescence indicates the nucleus of germ cells). Scale bar: 30 µm. **d.** *rbpl-1* RNAi in either soma or germline significantly reduces egg production. In the strain NL2098, RNAi is effective in the germline and intestine, while in the strain CB4856, RNAi is effective only in the soma. *P<0.05.

To explore the functions of RBPL-1, we first characterized the phenotype of the RBPL-1 mutant and found that homozygous *rbpl-1* mutation was embryonic lethal and the heterozygous worm shows wild type phenotype. Thus, in order to investigate the function of *rbpl-1* in adult worms, we used the RNA interference by bacteria feeding method. Plasmid sequencing confirmed the correct sequence of *rbpl-1* in the RNAi vector and RT-PCR showed that *rbpl-1* was effectively silenced in worms using this assay (Figure S1). To investigate the stage at which RBPL-1 functions, at 15°C we exposed worms to RNAi throughout the development and evaluated the worms in 12 h intervals. We found that the earlier RNAi was performed in the worm life cycle, the more severe were the defects in egg production. RNAi in adulthood partially inhibited egg production (Figure 2A). Moreover, all eggs produced by RNAi-silenced animals were dead before hatching. These results suggested that RBPL-1 is essential for various developmental processes ranging from embryonic development to gamete production.

### RBPL-1 Regulates Oocyte Production and Controls Germ Cell Proliferation

As shown in Figure 2A, downregulation of *rbpl-1* significantly reduced the number of eggs produced by the hermaphrodite. Therefore, we wondered whether RBPL-1 is indispensable for producing functional oocytes. The progeny of *rbpl-1* RNAi hermaphrodites crossed with *him-5* males, which supply normal functional sperm, were reduced compared with control RNAi hermaphrodites (Figure 2B), confirming that RBPL-1 is required for the generation of functional oocytes. Additionally, the strain AZ212 (*pie-1p::H2B::GFP*) in which GFP fluorescence is seen in the entire germline, enabled us to examine the morphological features of the germline. We found that *rbpl-1* RNAi worms had impaired germline development, reduced oocyte production, and multiple sets of chromosomes in some oocytes (Figure 2C), implying that loss of RBPL-1 may cause defects in oocyte production. We further investigated how RBPL-1 affected egg production using the strains NL2098 and CB4856. NL2098 is RNAi-sensitive in germline and in intestine, while CB4856 is RNAi-sensitive in soma only [14], [18], [19], [20]. In both strains, silencing *rbpl-1* significantly reduced fertility. Silencing germline- and intestine-derived *rbpl-1* led to embryonic lethality, while silencing somatically expressed *rbpl-1* produced progeny with a significantly reduced brood size (Figure 2D). These results indicated that both germline- and soma-derived RBPL-1 contribute to fertility, and that germline-expressed RBPL-1 plays a pivotal role during embryogenesis.

**Figure 3:**
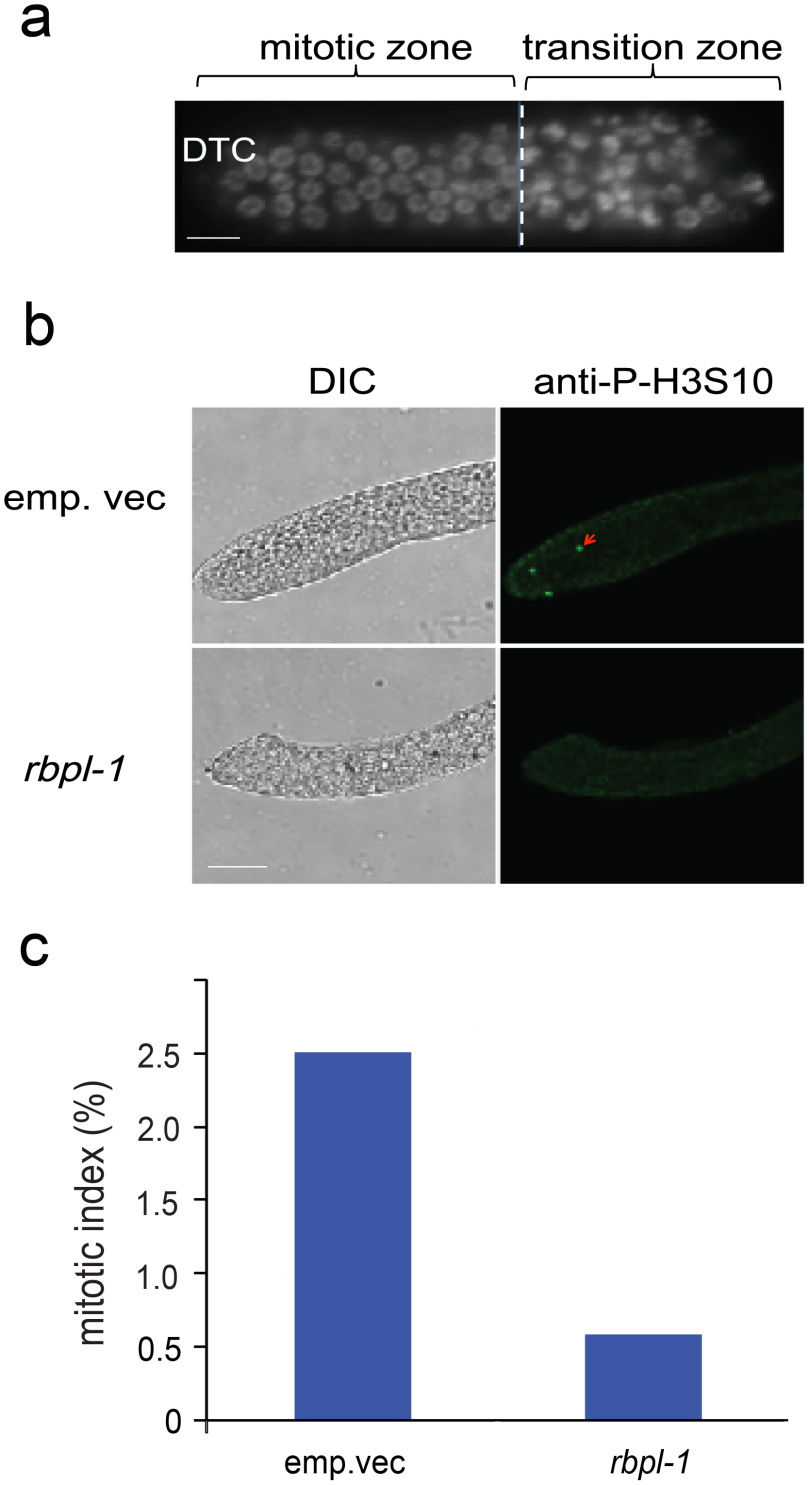
RNAi for *rbpl-1* causes defects of germ cell proliferation. a. The posterior end of the germline showing the mitotic and transition zones. Nuclei are labeled by H2B::GFP. Scale bar: 10 µm. **b.** Fewer mitotic cells are observed in *rbpl-1* RNAi worms than in control worms by immunofluorescence. The arrow indicates mitotic cells labeled with anti-P-H3S10. Scale bar: 10 µm. **c.** The mitotic index in *rbpl-1* RNAi worms is much lower than in control worms. Mitotic index: the number of cells in mitotic stage (staining by anti-P-H3S10) divided by the total cell number in mitotic zone (staining by DAPI).

Apart from the defects in egg production, we also found that the gonad of *rbpl-1* RNAi worms was smaller than that of the controls (data not shown), hinting that there may be a defect of germ cell proliferation. Within the *C. elegans* germline, cells in the mitotic zone keep dividing to sustain germ cell proliferation (Figure 3A). Therefore, we tracked cells undergoing mitosis using a mitosis marker (anti-P-H3S10) in this region, and found that there were fewer mitotically dividing cells in *rbpl-1* RNAi worms than in control worms (Figure 3B). These results were confirmed by analysis of the mitotic index [21]: the mitotic index was significantly lower in *rbpl-1* RNAi worms than in control worms (Figure 3C). Together, these data suggested that RBPL-1 regulates the proliferation of germ cells. We propose that depletion of RBPL-1 impairs germ cell proliferation, and ultimately impairs egg production.

### RBPL-1 Regulates Nutrient Synthesis in the Intestine

**Figure 4:**
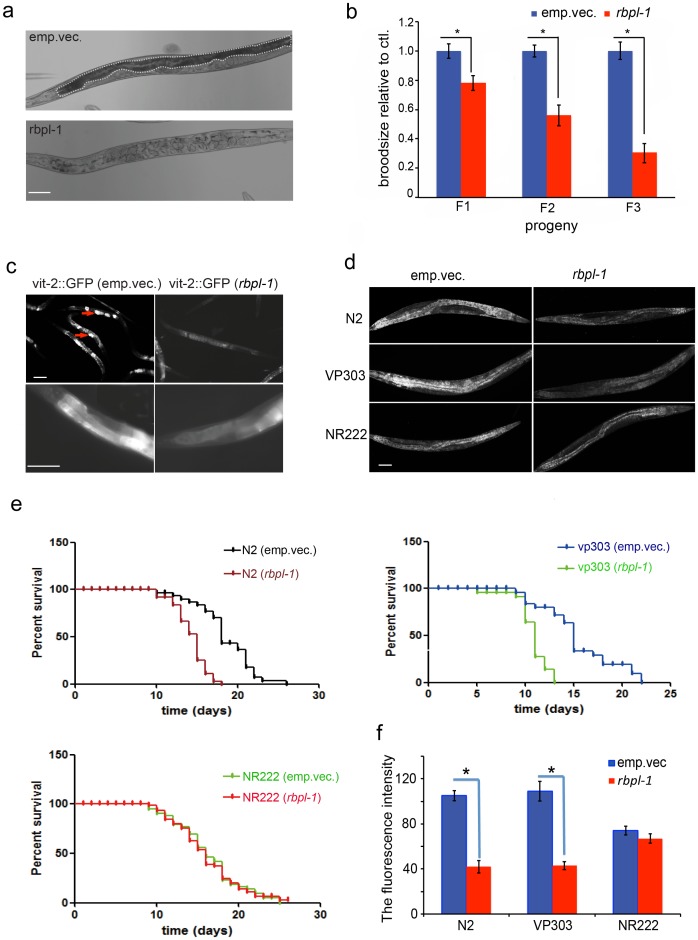
RNAi for *rbpl-1* results in defects of intestine development a. The intestine (indicated by dashed line) has nearly disappeared in the *rbpl-1* worms compared with controls. Scale bar: 25 µm. **b.** Continuous RNAi of *rbpl-1* in the intestine leads to a progressively reduced brood size through generations. *P<0.05. **c**. The production of vitellogenin (indicated by vit-2::GFP) is inhibited by RNAi silencing of *rbpl-1* (arrow head indicates the oocyte). Scale bar: 50 µm. **d.** Fluorescence imaging showing that lipid content is largely reduced upon silencing of *rbpl-1* in the whole worm (N2) or intestine only (VP303), but not in the hypodermis only (NR222). Scale bar: 25 µm. **e.** Survival time is shortened when silencing *rbpl-1* in the whole worm (N2) or only in the intestine (VP303), whereas RNAi for *rbpl-1* in the hypodermis (NR222) does not affect life span. **f.** Quantification of fluorescence intensity in Figure 4D indicates that the lipid content decreases significantly when silencing *rbpl-1* in the whole worm (N2), or only in the intestine (VP303), but not when restricted to the hypodermis (NR222). *P<0.01.

We have shown that down-regulation of *rbpl-1* in the soma results in reduced oocyte production (Figure 2D). Although it is not known how RBPL-1 functions somatically, we observed that *rbpl-1* RNAi worms were relatively sick and smaller than control worms. Owing to the fact that nutrient synthesis and translocation occurs in the intestine, and oocyte production requires this large supply of nutrients [22], we investigated whether RBPL-1 controlled intestine function or development. The evidence described below indicates that soma-derived RBPL-1 impacts intestine health and subsequently oocyte production. First, the body was much thinner and the intestine almost disappeared in the *rbpl-1* RNAi worms, suggesting a defect in nutrient synthesis, or the development of intestine (Figure 4A). Second, RNAi for *rbpl-1* in the strain VP303 (with an RNAi-sensitive intestine only) resulted in a significantly reduced brood size of F1 progeny compared with RNAi in controls, and continued RNAi further reduced the brood sizes of F2 and F3 generations (Figure 4B). Third, vitellogenin (marked by vit-2::GFP) synthesis was largely reduced when *rbpl-1* was silenced (Figure 4C). Fourth, Nile red lipid staining (Figure 4D) showed that the lipid content was remarkably reduced upon silencing of *rbpl-1* in the whole worm (N2) or only in the intestine (VP303) compared with the non-silenced controls. In contrast, RNAi for *rbpl-1* in the hypodermis (NR222, with RNAi-sensitive hypodermis only) did not affect lipid content. These results were also confirmed by quantification of the fluorescence intensities (Figure 4F). Fifth, worm lifespan, which relies heavily on the intestine [23], was found to be decreased when *rbpl-1* was silenced in the whole worm or only in the intestine, but not in the hypodermis (Figure 4E). Exogenous glucose partially restored lipid synthesis, and thereby increased the survival time for RNAi worms (Figure S2). Taken together, these findings suggest that RBPL-1 plays a vital role in controlling the development of the intestine and nutrient synthesis.

### RBPL-1, as a Nucleolus Protein, is Expressed in Diverse Tissues

**Figure 5:**
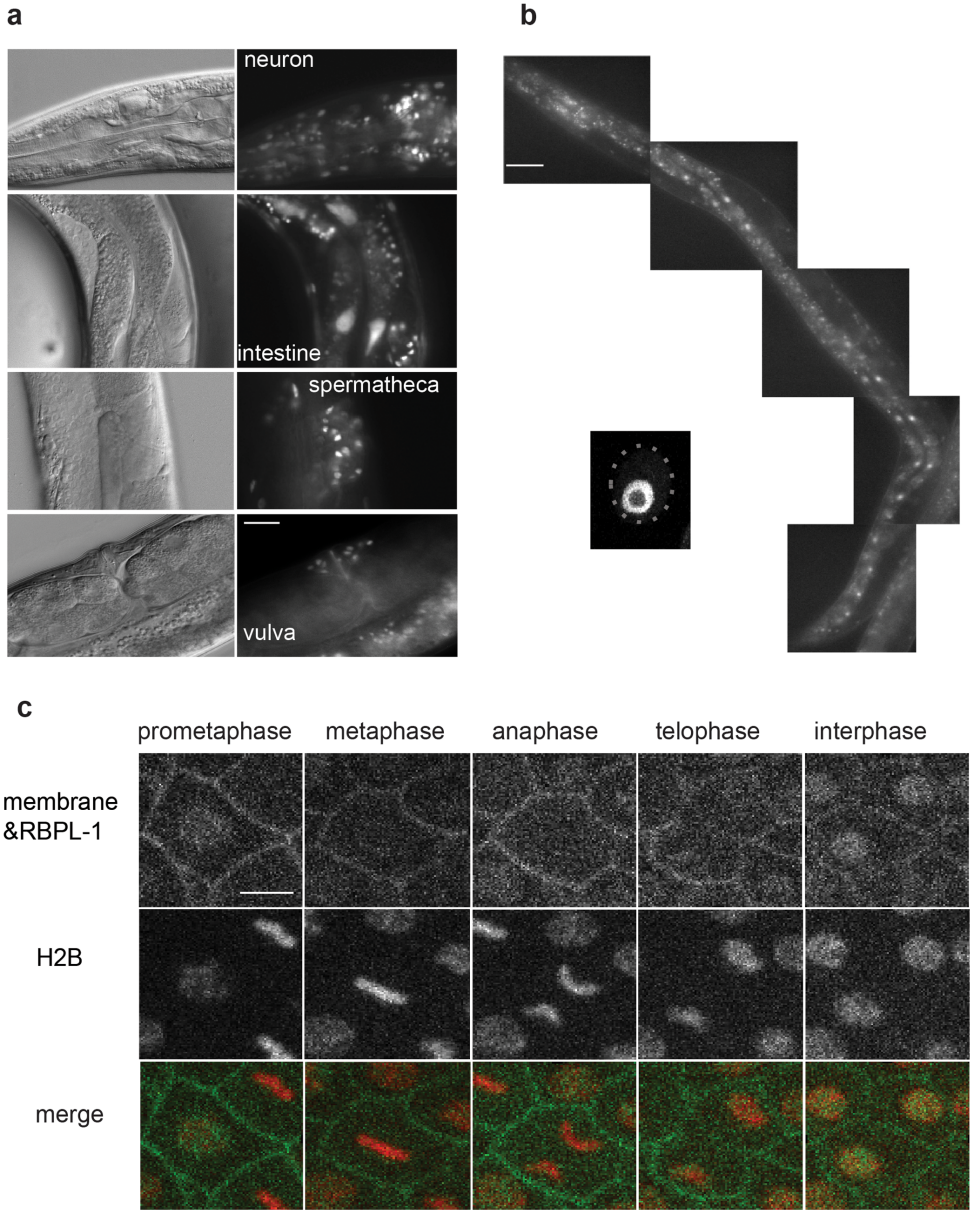
RBPL-1 is expressed in diverse tissues and localized to the nucleolus. a. RBPL-1::GFP is expressed in multiple tissues, including neurons, intestine, spermatheca, and vulva. Scale bar: 20 µm. **b.** RBPL-1::GFP is localized to the nucleolus of intestine cells. The bottom left image indicates the nucleolar localization of RBPL-1 within an intestine nucleus (the dashed line indicates the nucleus). Scale bar: 50 µm. **c.** Dynamics of RBPL-1 during embryogenesis. A transient disappearance of RBPL-1 fluorescence occurs from metaphase to telophase. Membrane and RBPL-1 are marked by GFP, and nuclei are shown by H2B::mCherry. Scale bar: 2 µm.

To examine the expression pattern and cellular localization of RBPL-1, we constructed a transgenic strain expressing a RBPL-1::GFP fusion protein driven by the *rbpl-1* promoter. RBPL-1 was expressed in a variety of tissues, such as the neurons, intestine, spermatheca, and vulva (Figure 5A), consistent with previous results (Figure 2) showing that RBPL-1 acts in both the germline and soma. RBPL-1 was highly expressed in the intestine (Figure 5B). Moreover, RBPL-1 was localized to the nucleolus (Figure 5B, the dashed oval indicates the nucleus zone), similar to the localization of its homologues in various other species [9]. As a control, *rbpl-1* RNAi for transgenic worms abolished the RBPL-1::GFP signal in the nucleus of embryo and intestine cells (Figure S3). By epifluorescence microscopy, we could detect RBPL-1 expression at the 30-cell stage (Figure S4). With a high-resolution spinning-disk confocal system, we observed the dynamics of RBPL-1 expression and localization during embryogenesis. Interestingly, the RBPL-1 signal disappeared as the cells entered metaphase and re-appeared as the cell exited metaphase (Figure 5C and Movies S1 and S2), indicating that RBPL-1 may be associated with cell mitotic division. This result, in combination with our previous ones showing that RBPL-1 in the germline affects embryo survival (Figure 3), suggests that RBPL-1, as a nucleolus protein, participates in cell division during embryogenesis.

### RBPL-1 may Act as a Cleavage and Polyadenylation Factor to Regulate Gene Expression

The homolog of RBPL-1 in fusion yeast, Mpe1, is a component of the protein complex cleavage and polyadenylation factor [12], and bioinformatic analysis (interactome on WormBase [24]) shows that most of the predicted RBPL-1-interacting proteins are cleavage and polyadenylation factors (Table 1). Thus, we proposed that if r*bpl-1* functions like its homologue in yeast as an mRNA cleavage and polyadenylation factor, numerous genes should be regulated by *rbpl-1*. Therefore, we performed microarray analysis comparing the embryos of *rbpl-1* RNAi worms and RNAi control worms. The results showed that 406 genes and 264 genes were downregulated and upregulated, respectively, by at least 10-fold, in the *rbpl-1* RNAi worms compared with the control worms (Table S1). Gene ontology analysis showed that these 670 genes encode a wide variety of cellular components, and are involved in a broad range of biological processes and molecular functions (Figure S5). Considering that RBPL-1 is located in nucleolus, we propose that RBPL-1 functions as an mRNA cleavage and polyadenylation factor to control the expression of a wide range of genes in *C. elegans*.

**Table 1:** List of polyadenylation factors predicted to interact with RBPL-1 according to the interactome on WormBase.

Gene	Gene ID	Interaction type	Gene description
***pcf-11***	R144.2	synthetic	cleavage and polyadenylation factor
***fipp-1***	F32D1.9	predicted	factor interacting with poly(A) polymerase
***cpsf-1***	Y76B12C.7	predicted	factor interacting with poly(A) polymerase
***clpf-1***	F59A2.4	predicted	cleavage/polyadenylation factor Ia subunit
***cpsf-3***	Y67H2A.1	predicted	cleavage and polyadenylation specific factor
***cpsf-4***	F11A10.8	predicted	cleavage and polyadenylation specific factor
***pap-1***	Y32F6A.3	predicted	a poly (A) polymerase

## Discussion

RBBP6 has been extensively studied in mammals, because it regulates the p53 pathway to suppress tumorigenesis and serves as a promising target for immunotherapy [7]. However, the homologs of RBBP6 in other species ranging from the unicellular parasite *Encephalitozoon cuniculi* to invertebrates all lack the Rb- and p53-binding domains [2]. This suggests that these two domains are newly evolved in mammals. In contrast, three other domains, namely the DWNN, Zinc knuckle, and RING finger domains, are highly conserved in all eukaryotic RBBP6s; however, their functions have attracted relatively less attention.

Here, we characterized RBPL-1, the only RBBP6 homolog in *C. elegans.* We showed that RNAi silencing of *rbpl-1* during the egg stage leads to embryonic lethality, while RNAi for *rbpl-1* in the adult stage inhibited oocyte production. Further examination of the gonad revealed that the knockdown of *rbpl-1* impairs germ cell proliferation. The observations that *rbpl-1* RNAi worms have fewer mitotic cells and that oocytes contains multiple sets of chromosomes indicate that RBPL-1 may control both mitotic and meiotic cell divisions in the germline. This result echoes that in mammalian cells where P2P-R, a RBBP6 isoform, has a potential role in mitosis [5], [9].

We show that RBPL-1 is not only indispensable for germline function, but it is also necessary in the soma. Our analysis indicates that the intestine is a major organ within which RBPL-1 acts. The intestine synthesizes and secretes yolk particles into the pseudocoelomic space (body cavity) that are ultimately taken up by the developing oocytes [25]. Depletion of *rbpl-1* affects nutrient synthesis in the intestine, and subsequently causes an insufficient nutrient supply for oocytes.

We also show that RBPL-1 is ubiquitously expressed in a variety of tissues. Notably, RBPL-1 is localized to the nucleolus, consistent with the presence of a nuclear localization signal at its C-terminus. Interestingly, RBPL-1 displays a transient disappearance at metaphase, suggesting that the dynamics of RBPL-1 may be involved in mitosis. This is consistent with our observations that down-regulation of *rbpl-1* leads to defects of germ cell proliferation. It should be noted that P2P-R also undergoes a dynamic change during mitosis, hinting that RBBP6 may have an evolutionarily conserved role in regulating the cell cycle.

Mpe1, the RBBP6 homolog in yeast, functions as a nucleolus mRNA cleavage and polyadenylation factor [12]. Our microarray analysis identified 670 genes that are affected by RBPL-1 during embryogenesis. In addition, many of the predicted interactors of RBBP6 play a role in mRNA cleavage and polyadenylation. Thus, we propose that RBPL-1, like yeast RBBP6, may act primarily as an mRNA cleavage and polyadenylation factor to regulate gene expression, and that its downregulation directly results in cell cycle dysfunction, leading to severe defects in the germline and soma. Further investigations of the RBPL-1-interacting proteins will help to elucidate the mechanisms by which RBPL-1 performs its roles.

## Supporting Information

Figure S1 **The** ***rbpl-1*** **mRNA level is remarkably reduced in** ***rbpl-1*** **RNAi worms compared with mock RNAi worms, indicating that RNAi is effective.**
(TIF)

Figure S2 **The survival rate is progressively increased by supplemental glucose (0–50 mM) to** ***rbpl-1*** **RNAi worms, but not to control ones.** (“n” indicates the total worm number counted).
(TIF)

Figure S3 ***rbpl-1*** **was effectively silenced in the embryo and intestine.** In control RNAi fed worms, GFP signals are detected in the nucleus of the embryo and intestine cells. In *rbpl-1* RNAi worms, GFP signals are depleted in the nucleus of embryos and intestine cells. The dotted fluorescence (outside the line) in intestine is autofluorescence.
(TIF)

Figure S4 **RBPL-1 is first expressed at approximately the 30-cell stage, as shown in the middle paired panels.**
(TIF)

Figure S5 **The genes regulated by** ***rbpl-1*** **control a broad range of pathways.**
(TIF)

Table S1 **List of upregulated and downregulated genes by at least 10-fold in the embryos of** ***rbpl-1*** **RNAi worms as compared with by microassay analysis.**
(XLSX)

Movie S1 **Dynamics of RBPL-1 during embryogenesis.** Plasma membrane and RBPL-1 are marked with GFP, and nuclei are shown by H2B::mCherry. RBPL-1 (GFP in nucleus) disappears as the cell enters metaphase and re-appears as the cell exits metaphase; in contrast, H2B (mCherry) exists at every stage.
(MOV)

Movie S2 **A close-up of one cell from Movie S1.**
(MOV)
