# Supplementary figures and images for: The C. elegans Homolog of RBBP6 (RBPL-1) Regulates Fertility through Controlling Cell Proliferation in the Germline and Nutrient Synthesis in the Intestine
Source: PLoS One. 2013 Mar 11;8(3):e58736. doi: 10.1371/journal.pone.0058736 (PMC3594146; doi:10.1371/journal.pone.0058736)

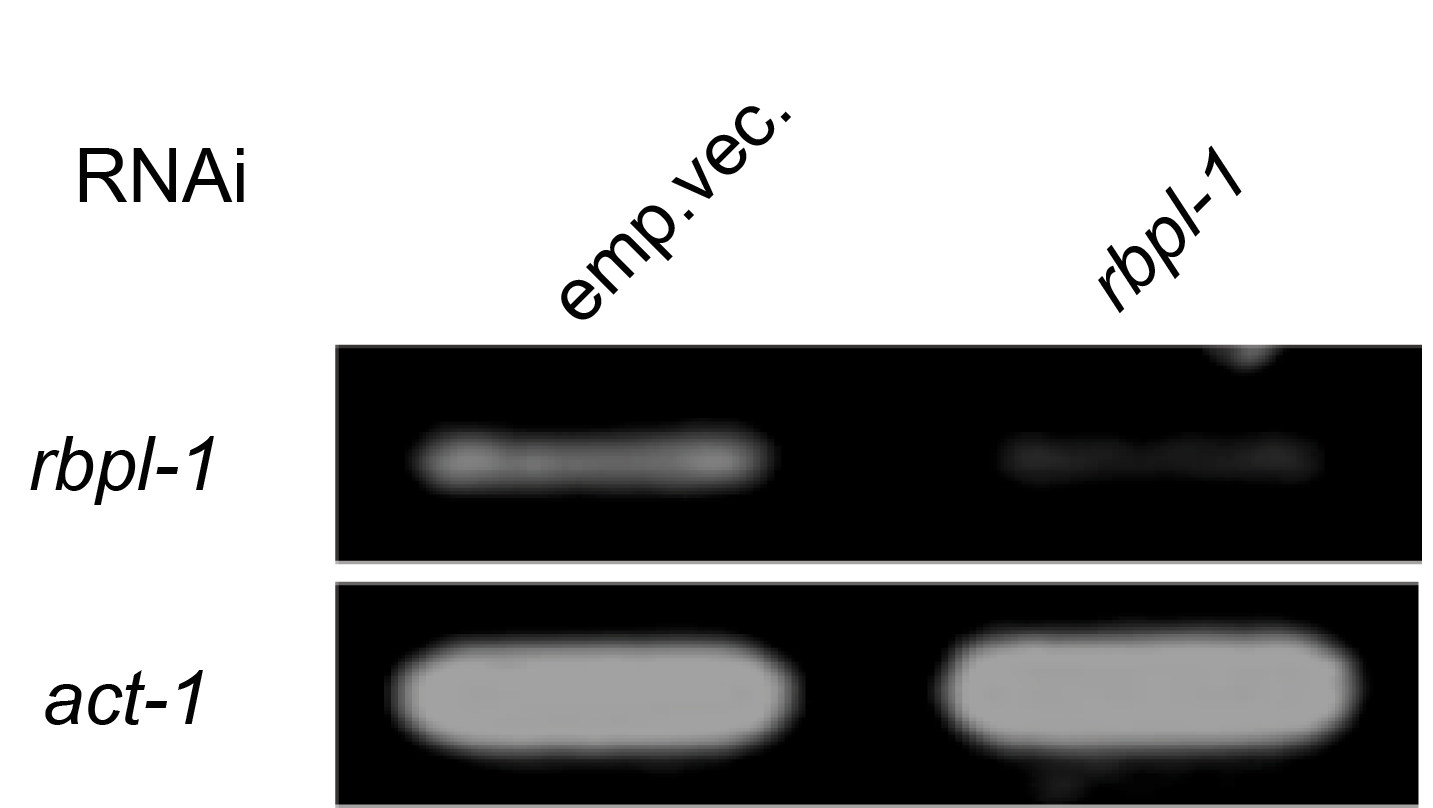

Supplement: Figure S1 — The rbpl-1 mRNA level is remarkably reduced in rbpl-1 RNAi worms compared with mock RNAi worms, indicating that RNAi is effective. (TIF) [file pone.0058736.s001.tif]

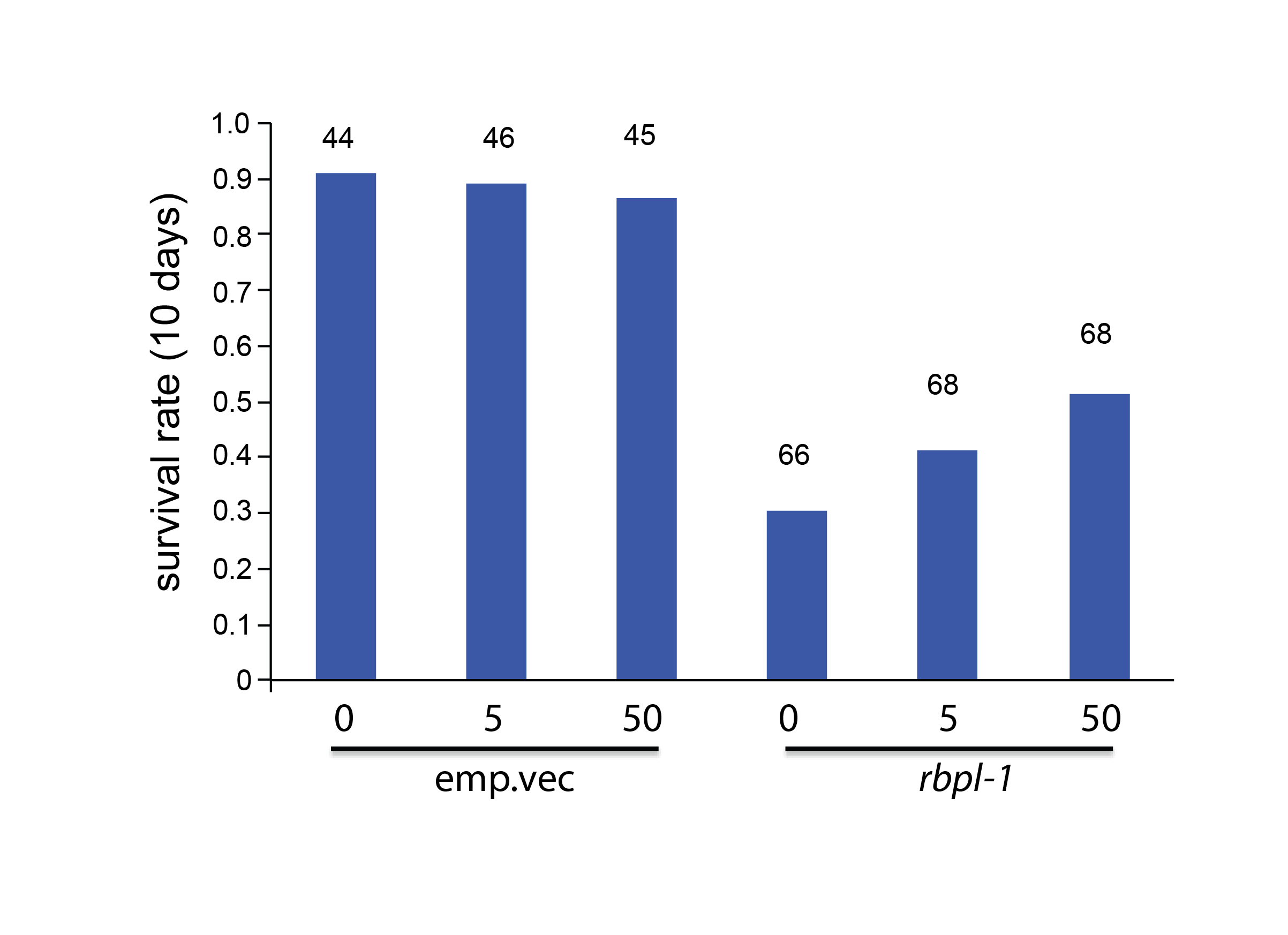

Supplement: Figure S2 — The survival rate is progressively increased by supplemental glucose (0–50 mM) to rbpl-1 RNAi worms, but not to control ones. (“n” indicates the total worm number counted). (TIF) [file pone.0058736.s002.tif]

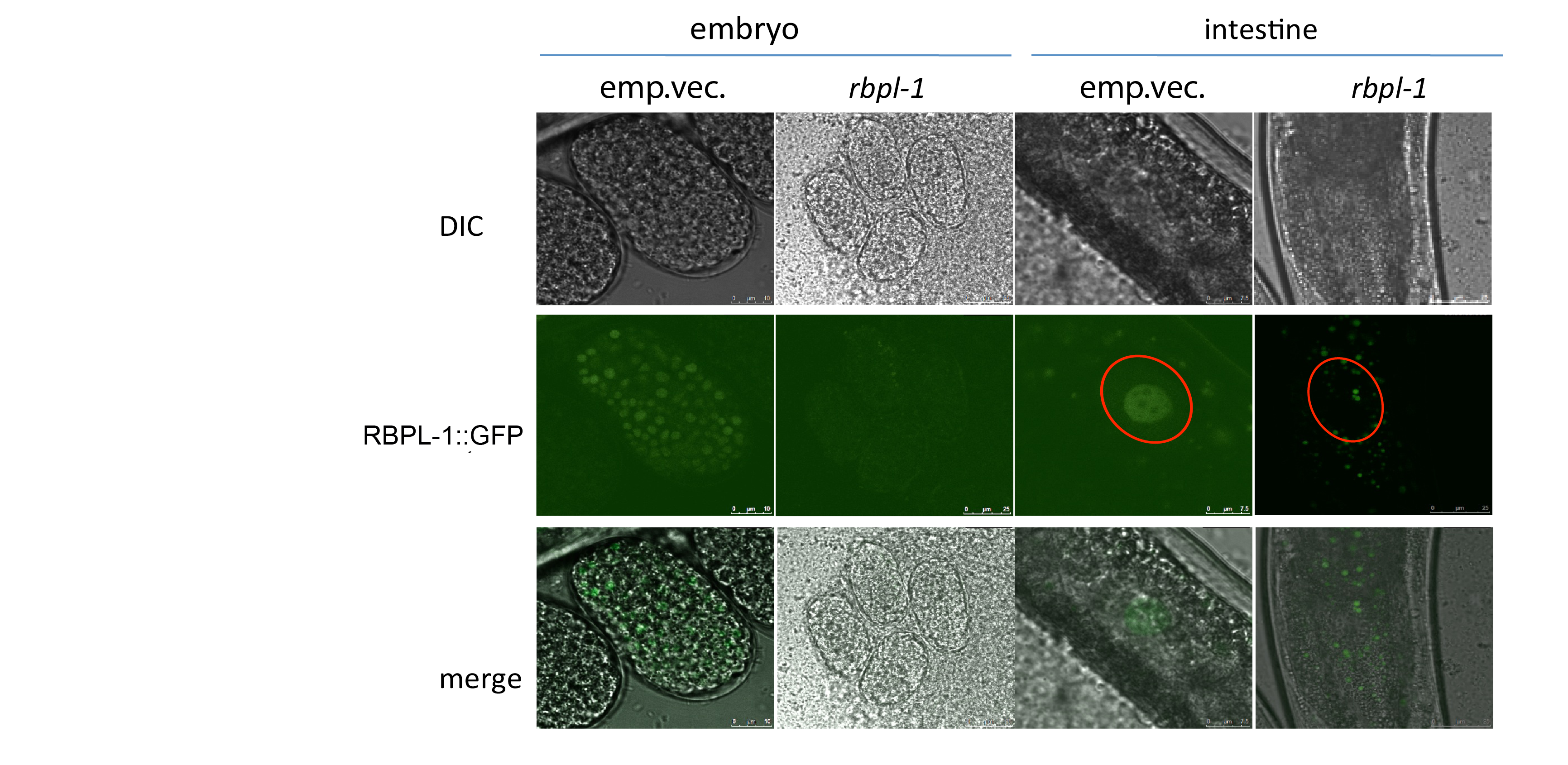

Supplement: Figure S3 — rbpl-1 was effectively silenced in the embryo and intestine. In control RNAi fed worms, GFP signals are detected in the nucleus of the embryo and intestine cells. In rbpl-1 RNAi worms, GFP signals are depleted in the nucleus of embryos and intestine cells. The dotted fluorescence (outside the line) in intestine is autofluorescence. (TIF) [file pone.0058736.s003.tif]

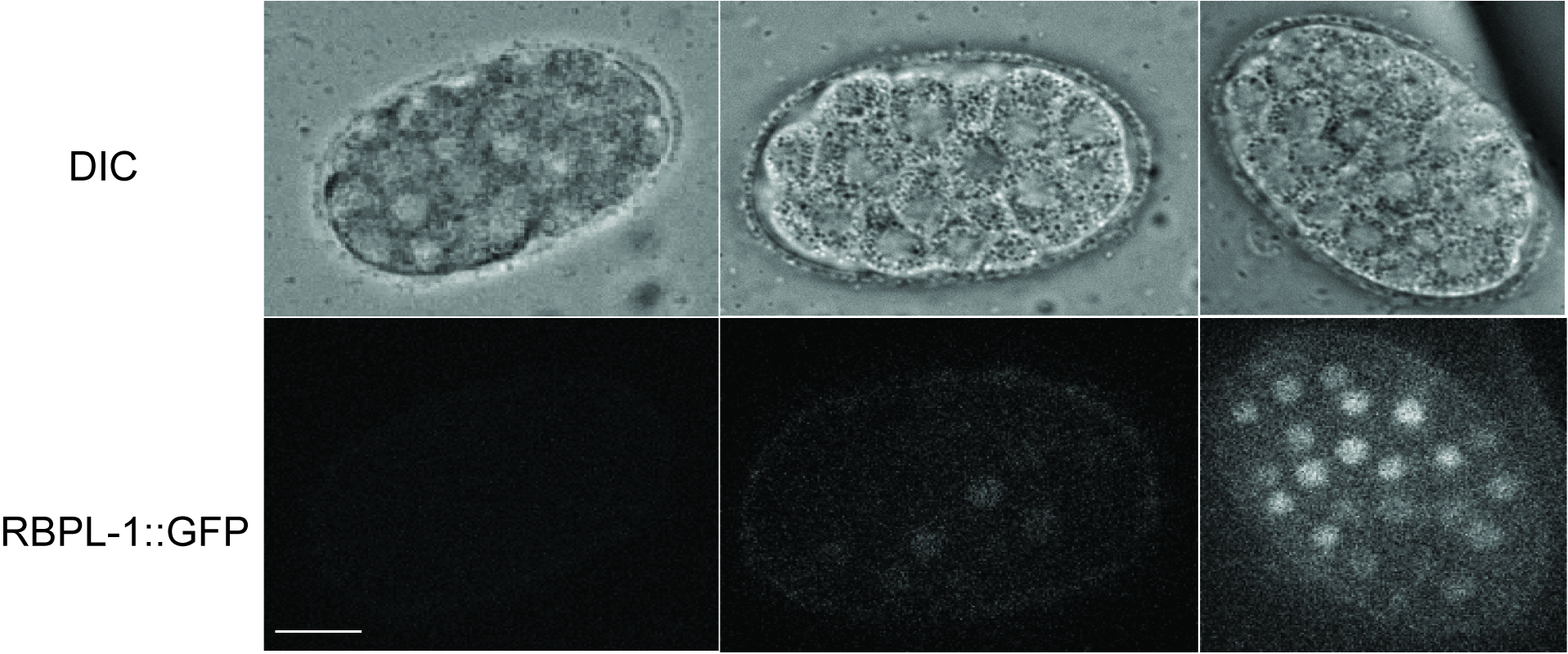

Supplement: Figure S4 — RBPL-1 is first expressed at approximately the 30-cell stage, as shown in the middle paired panels. (TIF) [file pone.0058736.s004.tif]

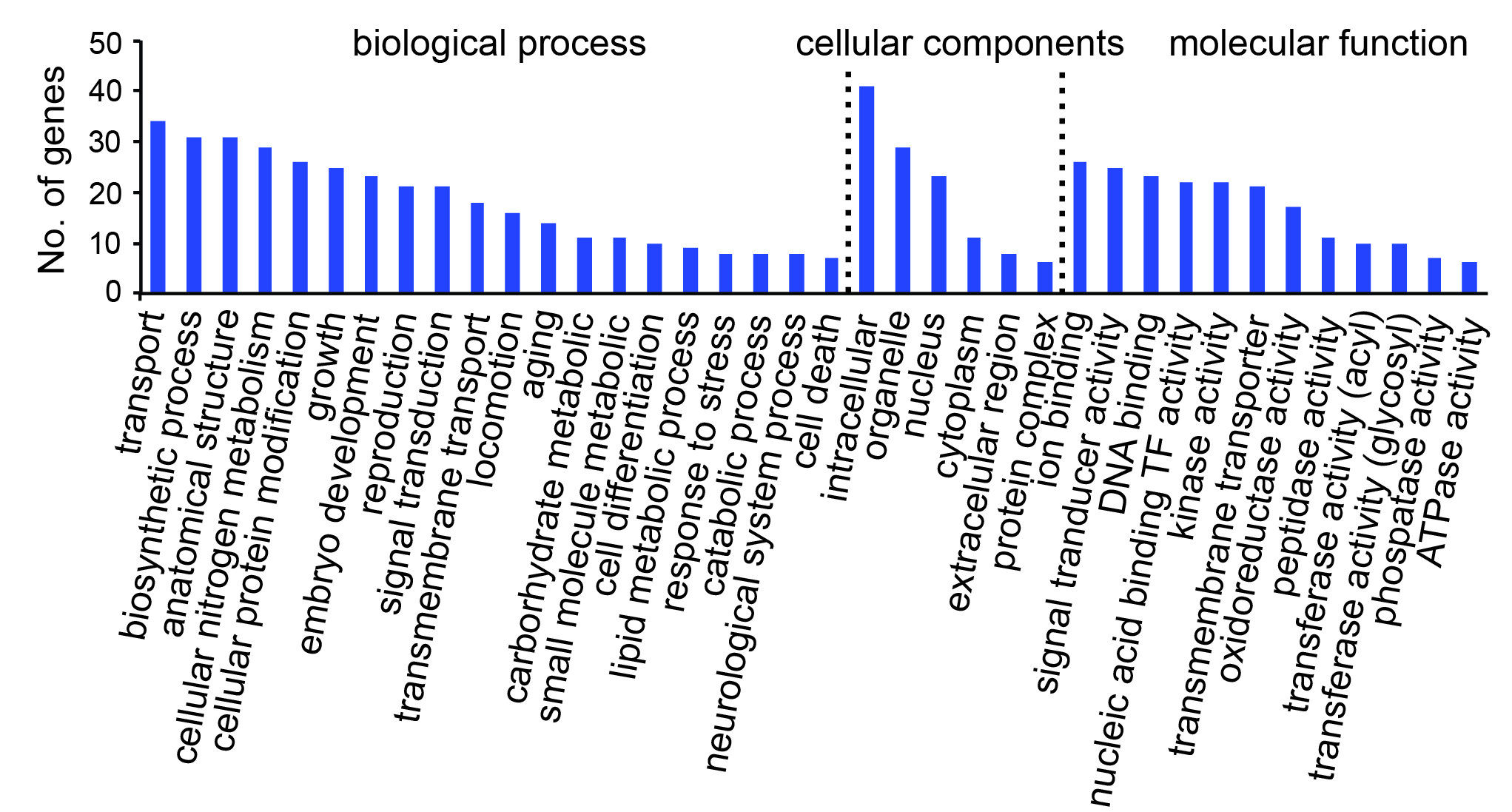

Supplement: Figure S5 — The genes regulated by rbpl-1 control a broad range of pathways. (TIF) [file pone.0058736.s005.tif]
